# Exosomes of human adipose stem cells mitigate irradiation injury to salivary glands by inhibiting epithelial-mesenchymal transition through miR-199a-3p targeting Twist1 and regulating TGFβ1/Smad3 pathway

**DOI:** 10.7150/thno.102346

**Published:** 2025-01-02

**Authors:** Xiaotong Guo, Zhu Huang, Fan Wu, Wentao Jiang, Yiyang Li, Tao Wang, Simon D Tran, Zhengmei Lin, Xinyun Su

**Affiliations:** 1 Hospital of Stomatology, Guangdong Provincial Key Laboratory of Stomatology, Guanghua School of Stomatology, Institute of Stomatology, Sun Yat-sen University, Guangzhou, Guangdong, 510055, China.; 2 McGill Craniofacial Tissue Engineering and Stem Cells Laboratory, Faculty of Dental Medicine and Oral Health Sciences, McGill University, Montreal, QC H3A0C7, Canada.; 3 School of Stomatology, Shandong Second Medical University, 261053, China.

**Keywords:** exosomes, salivary glands, radiation, stem cell, epithelial-mesenchymal transition

## Abstract

**Rationale:** Currently, irradiation-injured salivary glands (IR-SG) lack effective clinical treatment options. Emerging treatments using exosomes (Exo) have shown promising outcomes for various diseases. However, the efficacy of exosome in treating IR-SG remains unexplored. This study aimed to use exosomes to restore IR-SG function and to explore their underlying mechanisms.

**Methods:** Exosomes isolated from human adipose-derived stem cell (ADSC-Exo) were injected into C57BL/6 mice that had their salivary glands injured with 14Gy. RNA sequencing profiled differentially expressed miRNAs and mRNAs of IR-SG. Epithelial-mesenchymal transition (EMT) mechanisms were further examined using SMG-C6 cells.

**Results:** Exo-treated mice had a 96% increase in saliva secretion, higher cell proliferation, upregulated tissue repair/regeneration genes, and preserved functional cells with fewer collagen fibers compared to saline-treated mice. Exo treatment increased the expression of epithelial cell markers while decreasing mesenchymal cell markers. Notably, miR-199a-3p was significantly upregulated in Exo-treated mice, promoting cell growth and reducing EMT. Twist1, an EMT transcription factor, was identified as a direct target of miR-199a-3p and confirmed by luciferase assays. Twist1 overexpression promoted EMT, but Exo treatment or Twist1 knockdown reduced EMT marker expression and inactivated the TGFβ1/Smad3 pathway.

**Conclusions:** ADSC-Exo is a promising therapy for IR-SG, primarily by mitigating EMT through miR-199a-3p targeting Twist1 and regulating the TGFβ1/Smad3 pathway.

## Introduction

Radiotherapy (RT) is commonly used to treat head and neck cancer, effectively targeting tumors but also affecting nearby normal oral tissues, which can lead to salivary gland (SG) hypofunction. Current clinical treatments for RT-induced SG hypofunction remain palliative and preventive. Chronic hyposalivation is due, in part, to the SG's inability to regenerate functional cells and the development of fibrosis 1, 2. Significant fibrotic damage has been observed in SGs following irradiation (IR) in mice, minipigs, and humans 1-5. Epithelial-to-mesenchymal transition (EMT) is a process where epithelial cells, such as acinar cells, lose their epithelial characteristics and acquire mesenchymal phenotypes and behavior. EMT is divided into three subtypes based on biological context 6. Type 1 occurs during embryonic development, while Type 3 is involved in cancer progression when genetic changes, such as in oncogenes and tumor suppressor genes, drive tumor growth and metastasis. Type 2 EMT, distinct from the other two, happens during wound healing and tissue repair but can lead to fibrosis and contribute to the hypofunction of irradiated SG 2, 6. Initially, Type 2 EMT aids in repairing IR-injured SG (IR-SG) by generating fibroblasts and other related cells, but excessive repair can cause fibrosis and SG dysfunction. Transforming growth factor β1 (TGFβ1) plays a crucial role in inducing EMT in various epithelial cell types in salivary glands 7, 8 and is considered a primary driver of EMT 9. The TGFβ/Smad signaling pathway promotes inflammation, EMT, and abnormal extracellular matrix deposition, which contribute significantly to organ fibrosis 6, 10, 11. Inhibition of TGFβ1 has shown potential in reducing IR-induced fibrosis in the lung and rectum 2, 12, 13 and duct ligation-induced SG fibrosis in mouse models 14. Twist1, a transcription factor (TF), is an important regulator of IR-induced EMT (IR-EMT) and fibrosis 15. Twist1 activation induces EMT, elevates TGFβ expression, and may promote fibrosis, as demonstrated in a hepatitis mouse model 9. However, research on IR-EMT in IR-SG remains limited, and the specific interactions among TGFβ1, Twist1, and fibrosis in IR-induced SG damage are still not fully understood.

Exosomes (Exo) are crucial for intercellular communication, transferring bioactive molecules (such as microRNAs (miRNAs) and proteins) to target cells, regulating cellular functions and mediating cell-to-cell crosstalk. They represent a promising avenue for cell-free therapy in several diseases, including myocardial injury 16, IR-induced lung and intestinal damage 17, 18 and autoimmune diseases 19. Exosome treatments have been shown to enhance cell proliferation, reduce apoptosis, modulate the immune system, induce tumor cell death, and alleviate oxidative stress 20-22. Additionally, exosomal miRNAs have demonstrated effectiveness in alleviating fibrosis in myocardial, renal, and liver tissues 23-25 and can reverse EMT in radiation-induced lung injury by influencing gene expression in recipient cells 17. Consequently, extracellular vesicles/exosomes from adult stem cells hold potential for SG regeneration, particularly in promoting angiogenesis and neurogenesis 26. However, the application of exosomes in SG research remains limited. For instance, only a few studies have explored their therapeutic effects in Sjögren's syndrome 27-30. Additionally, the use of exosomes in IR-SG is notably limited. Specifically, exosomes derived from SG organoids have been shown to stimulate epithelial growth, mitosis, and neuronal growth in IR-injured SG *ex vivo* 31. A recent study found that urine-derived stem cell exosomes could repair acute IR-SG damage during a short one-week observation 32. Despite these findings, it is still unclear whether mesenchymal stem cell-derived exosomes can attenuate hypofunction of IR-SG *in vivo*, and their therapeutic mechanisms remain unknown. Thus, it is worth applying the exosomes to treat IR-SG and elucidate the effect and the mechanism of the Exo treatment.

In this study, we tested the therapeutic effect of human adipose stem cell-derived exosomes injected in mice that had their SG injured with IR and explored their mechanisms of action. We found that exosome treatment protected functional cells in SGs, mitigated EMT and fibrosis, and restored the salivary function in IR-injured SGs. Additionally, we identified that miR-199a-3p in the exosomes might be one of the primary factors attenuating EMT in IR-injured SG by targeting Twist1 and regulating TGFβ1/Smad3 signaling pathway. As a secondary aim, we found that soluble proteins derived from conditioned media (CM) were unable to restore SG function post-IR. Together, our study enhances the understanding of the mechanisms underlying IR-induced EMT in SG and indicated that exosomes as a promising cell-free therapy for restoring function of IR-SG. This effect is achieved, in part, by reducing EMT in IR-SG via the miR-199a-3p/Twist1/TGFβ1/Smad3 pathway.

## Methods

### Isolation of human adipose stem cells

Human adipose tissues (n = 5, aged 30-76, 3 males and 2 females) were isolated from flap grafting surgeries in the Hospital of Stomatology of Sun Yat-Sen University (ethical approval number: KQEC-2024-103-01). Adipose-derived stem cells (ADSCs) were isolated and expanded as described in our previous study 33. In brief, after three washes with PBS, tissues were minced into small pieces and incubated with 0.75% collagenase Type I for 30 min at 37 °C on a shaker. After centrifugation at 2,000 g for 5 min, the pelleted ADSCs were resuspended with PBS and filtered through a 70 μM cell strainer (BD Falcon, Bedford, MA). Then, the cells were cultured in alpha-minimal essential medium supplemented with 10% fetal bovine serum (FBS; 1009914, Thermo Fisher Scientific, USA), and 100 U/mL penicillin/streptomycin (15140122, Thermo Fisher Scientific, USA). Cells from passages 3-6 were used for the experiments.

### Preparation and identification of exosomes

ADSCs were cultured until passages 3-6. After washing with PBS, serum-free medium was added to the cells, and the culture medium was collected after 48 hours of incubation. Exosomes were isolated using ultracentrifugation-based techniques 34. In brief, the conditioned medium was centrifuged at 300 g for 10 min and at 2,000 g for 15 min to eliminate dead cells and cell debris. Then, a 30 min centrifugation at 10,000 g was performed to remove large microvesicles and apoptotic bodies. The supernatant was filtered using a 0.22 μm filter and then centrifugated at 120,000 g for 90 min. The exosome fraction (located in the pellet) was resuspended in 100 μL PBS and used for experiments. The soluble protein-rich fraction (SP) was collected from the supernatant after the 120,000 g ultracentrifugation step and was concentrated using a Millipore ultrafiltration centrifugal tube (3 kDa).

The particle size distribution of the exosomes was tested by nanoparticle tracking analysis (NTA) with a Nano Sight NS300 (Malvern, Worcestershire, UK). The morphology of ADSC-Exo was observed using transmission electron microscopy (TEM, Japan). The concentration of Exo, CM or SP was measured by a BCA (bicinchoninic acid) assay kit (Thermo Fisher Scientific, Rockford, IL). Western blot analysis was performed to examine exosomal markers, including CD9, tumor susceptibility gene 101 (TSG101), CD81, and Calnexin (negative control, Abcam, UK).

### Animals and irradiation

All animal experiments were approved by the Laboratory Animal Center of Sun Yat-sen University (ethical approval number: SYSU-IACUC-2021-000053) and animal experiments conform to ARRIVE guidelines. 6-8 weeks old C57BL/6 female mice were obtained from the National Resource Center of Model Mice (Nanjing, China), and kept in clean conditions with food and water in the animal resource center at Sun Yat-sen University. Mice were anesthetized with 1% pentobarbital sodium. 14 Gy irradiation was precisely slit-collimated to target the salivary glands (head and neck area) using the RS 2000 small animal irradiator, with accuracy ensured by a custom-designed transparent restrainer. Three lead blocks were strategically placed to shield non-target areas. All mice were randomly divided into 5 groups (with 6 mice per group): (1) Sham IR group (no irradiation, no injection); (2) NS group (IR + normal saline (NS) injection); (3) CM group (IR + ADSC-derived CM (ADSC-CM) injection); (4) Exo group (IR + ADSC-Exo injection); (5) SP group (IR + ADSC-SP injection). According to their groups, mice were injected with either 100 μL NS, ADSC-CM, SP or Exo (2 µg/µL) through their tail vein at 5 to 7 days post-irradiation, once a week for two consecutive weeks. Mice were sacrificed either at 8 weeks or 16 weeks post-IR.

### Salivary secretory function measurements

10 μL/g body weight of pentobarbital sodium was used as the anesthetic to measure salivary flow rate (SFR) of mice at weeks 0, 4, 8, 12, and 16 post-IR. As previously described 35, stimulated saliva was collected for 10 min following the injection of 0.5 mg/kg body weight of pilocarpine (Sigma-Aldrich, ST. Louis, USA). The weight of the collected saliva was considered equivalent to its volume, assuming a density of 1 g/mL. Additionally, the lag time prior to saliva secretion was recorded to assess salivary gland function. The total protein of saliva was measured by the BCA assay kit.

### H&E, PAS, and Masson's trichrome staining

Specimens were fixed in 4% paraformaldehyde (P6148, Sigma-Aldrich), embedded in paraffin, and sectioned into 6-8 μm thick slices. These slices were stained with Hematoxylin and Eosin (H&E). Periodic Acid-Schiff (PAS) staining was conducted using a PAS Staining Kit (Servicebio, G1008, China) according to the manufacturer's instructions. Masson's trichrome staining kit (Solarbio, G1340, China) was used to assess tissue fibrosis according to the manufacturer's instructions. 5-8 representative fields were randomly selected for analysis. The percentage of the surface area occupied by acinar cells relative to the entire tissue area and the collagen-rich regions were quantified using NIH Image J software.

### Hydroxyproline assay

To quantify total collagen in the SMG, a Hydroxyproline Assay Kit (Solarbio, China) was used according to the manufacturer's protocol. In brief, 10 mg of SMG tissue was digested with 37% hydrochloric acid by boiling for 10 min, and the supernatant was collected after centrifugation at 16,000 rpm for 20 min. The optical density (OD) was measured using a microplate reader (BioTEK, UK) at 560 nm. Three independent experiments were performed.

### Transmission electron microscopy (TEM)

SG specimens were fixed in 2.5% glutaraldehyde in 100 mM phosphate buffer with 2% PFA immediately after being isolated from the mice and minced into small pieces. The samples were then post‑fixed in 1% osmium tetroxide, dehydrated through a graded ethanol series, cleared in acetone, and embedded in Eponate 12 resin (Ted Pella Inc., Redding, USA). Ultrathin sections (70 nm thick) were cut with a diamond slicer (Diatome, Nidau, Switzerland) on a Leica EM UC7 ultramicrotome (Leica Microsystems GmbH, Wetzlar, Germany). The sections were stained with a 2% uranium acetate solution and a 2.6% Lead citrate solution, and observed on a transmission electron microscope (HITACHI, MA, USA) operated at 80 kV.

### Immunohistochemical staining

To detect cell proliferation in SG tissues, immunohistochemical staining was conducted. Samples in paraffin were sliced into 6-8 μm-thick sections. After deparaffinization and rehydration, heat-induced antigen retrieval was performed for 30 min at 95 °C with 10 mM Citrate Buffer solution (pH 6.1) and cooled down for 30 min at room temperature. Then, the immunohistochemistry (IHC) process was performed following the manufacturer's instructions. Briefly, slices were blocked with 10% goat serum for 1 h at room temperature. The primary antibody, rabbit anti-Ki67 (1:400, AF0198-50, Affinity, USA) or PBS (negative control) was applied overnight to the SG tissues at 4 °C. Then slices were incubated with a secondary antibody (1:200, Goat anti-rabbit-IgG (2268327, Invitrogen, USA) for 1 h at room temperature. DAB (GK600705, Gene Tech, China) was incubated with SG tissues for 3 min, followed by rinsing in water. Then hematoxylin staining was performed followed by dehydration in an ethanol series. Positive cells in eight fields per gland were quantified using Image J software.

### Immunofluorescent (IF) staining

SG tissues were embedded in optimal cutting temperature (OCT) and sectioned into 6-8 µm thick sections. After fixing with 4% paraformaldehyde for 15 min, slides were blocked with 10% donkey serum for 1 h at room temperature. Primary antibodies were as follows: rabbit anti-aquaporin 5 (1:200, AQP5, Novus, USA), mouse anti-alpha smooth muscle actin (1:200, α-SMA, af1032, Affinity), and rabbit anti-cytokeratin 5 (1:400, CK5, Abcam, USA); PBS served as a negative control. The SG tissues were incubated overnight with primary antibodies or PBS at 4 °C. After washing with PBS, slides were incubated for 1 h with a secondary antibody (1:200), specifically, Goat anti-rabbit Alexa Fluor® 594-conjugated. 4, 6-diamidino-2-phenylindole, dihydrochloride (DAPI; D9542, Sigma, USA) was utilized to label the cell nuclei. Six representative images were captured for each sample using a Leica DM4000 fluorescent microscope, and the fluorescence signal intensity was analyzed using ImageJ software (NIH).

### Quantitative reverse transcription polymerase chain reaction (qRT-PCR)

Total RNA was extracted from the SGs using TRIZOL reagent (15596018, Invitrogen, Carlsbad, USA). First-strand cDNA synthesis was performed using the High-Capacity cDNA Reverse Transcription Kit (RR036A, TAKARA, Japan) with 50 ng of RNA per sample. Small RNA was extracted according to the manufacturer's instructions (Accurate Biology, China). PCR was conducted on a LightCycler 96 system (Roche, Sweden). The primers used in this study were MMP2, NGF, EGF, Sox2, FGF2, VEGF, IGF1, BMP7, Sox10, AQP5, E-cadherin, Collagen1, Twist1, CD44, Snail1, TGFβ1, Smad3, miR-199a-3p and miR-490-5p. Glyceraldehyde-3-phosphate dehydrogenase (GAPDH) served as the endogenous reference. U6 and miR-484 were used as the internal control for microRNA expression analyses 36. Results were expressed as fold changes in relative gene expression.

### RNA sequencing

RNA integrity was determined using an Agilent 2100 Bioanalyzer (Thermo Fisher Scientific, Waltham, MA, USA), and samples with an RNA integrity number (RIN) ≥ 7 were selected for further experiments. Raw data were filtered using SOAPnuke (v1.5.6). Libraries were created, and DNA nanoballs (DNBs) containing multiple DNA copies were generated. Clean reads were mapped to the mouse reference genome using Hisat2 (v2.1.0) with default settings. Gene expression quantification was calculated using featureCounts (v1.6.4). The GO terms and KEGG Pathway analysis were performed on the most differentially expressed mRNAs, selected based on criteria of |log_2_FC| > 1 and p < 0.05.

Small RNA sequencing was performed using the BGISEQ-2000 platform. Sequencing libraries were constructed, and raw data were converted to FASTQ format. Read quality was assessed to remove adapter dimers, junk, low complexity sequences, common RNA families (rRNA, tRNA, snRNA, snoRNA), and repeats. Unique sequences of 18-26 bases were mapped to species-specific precursors in miRBase 22.0 by BLAST search to identify known and novel 3p- and 5p-derived miRNAs. Differentially expressed miRNAs were identified using criteria of |log_2_FC| > 1 and p < 0.05. MiRNAs with significant expression differences between NS and Exo groups were displayed in a heat map based on miRBase database entries.

### Irradiation model *in vitro*

The submandibular gland epithelial C6 (SMG-C6) cell line was originally derived from rat submandibular acinar cells and established by Quissell *et al.* 37, 38. Cells were cultured in DMEM/F12 medium (C11330500CP, Thermo Fisher Scientific, USA) supplemented with 2.5% FBS, 1% Penicillin-Streptomycin, 200 nM L-glutamine (G0200, Solarbio, China), 5 μg/mL insulin (HY-P1156, MCE, China), 80 ng/mL epidermal growth factor (EGF; PeproTech, USA), 1.1 µM hydrocortisone (G8450, Solarbio, China), 0.03 µg/mL retinoic acid (A9120, Solarbio, China), 2 nM triiodothyronine (HY-A0070, MCE, China), and 5 mg/mL transferrin (T8010, Solarbio, China) in a humidified atmosphere with 5% CO_2_ at 37 °C. Cells were seeded in 6-well plates and divided into three groups for testing the exosome treatment effect: (1) Sham IR group (no irradiation, no treatment); (2) NS group (IR + normal saline); (3) Exo group (IR + ADSC-Exo). Irradiation at 9 Gy was performed using the RS2000 irradiator with a reflector when cells reached 70% confluence. After irradiation, ADSC-Exo (2 μg/mL) was diluted in normal saline and administered to the cells according to the experimental groups.

### Exosome and miRNA localization

Dil (C7001, Thermo Fisher Scientific) was utilized to visualize the uptake of exosomes by the cells. The staining process was performed following the manufacturer's instructions. Briefly, 0.2 μL Dil was incubated with the isolated exosomes at room temperature for 20 min. After washing with PBS, the exosomes were centrifuged at 120,000 g for 90 min. Dil-labeled exosomes were collected from the pellet and incubated with SMG-C6 cells for 12 h. Cells were fixed in 4% paraformaldehyde for 30 min and subsequently stained with F-actin ((1:100, filamentous actin, Beyotime, China), and 4′, 6diamidino-2-phenylindole (5 mg/mL, DAPI, Beyotime, China). The uptake of exosomes was visualized by confocal microscopy (Olympus, FV3000, Japan).

To confirm the encapsulation of miR-199a-3p within exosomes, the ADSC-conditioned medium was treated with RNase I (2 mg/mL) or a combination of RNase I and Triton X-100 (0.1%) for 30 min. RNase I was used to deplete free extracellular miRNAs that are not in proteins or EVs, while Triton X-100 was used to disrupt membrane components. Additionally, to verify that miR-199a-3p is specifically encapsulated within exosomes, GW4869 (10 μM, MCE, China), an exosome secretion inhibitor, was administered to ADSCs for 48 h.

For direct visualization of the delivery of exosomal miR-199a-3p, ADSCs were transfected with 100 nM of Cy3-labeled miR-199a-3p mimics (constructed by Gene Pharma, China) and incubated for 48 h. Exosomes were subsequently isolated using ultracentrifugation and co-cultured with SMG-C6 cells for 6 h. To confirm the bioactive function of miR-199a-3p within the exosomes, Cy3-labeled mimics, and Hoechst-stained nuclei were observed using confocal microscopy (Olympus, FV3000, Japan).

### Cell proliferation

Cell growth was assessed with the Cell Counting Kit-8 (CCK-8; Dojindo, Japan). 1,000 SMG-C6 cells were seeded into each well of a 96-well plate. 10% (v/v) CCK-8 solution was added to the cells and incubated for 2 h. The optical density (OD) was subsequently measured by a microplate reader (BioTEK, UK), and three independent experiments were carried out.

### Cell transfection

IR-injured SMG-C6 (IR-SMG-C6) cells were transfected with si-Twist1 (Genecefe, China), mature rno-miR-199a-3p mimic (Ribo, Guangdong, China), rno-miR-199a-3p inhibitor (Ribo, China), or the corresponding negative controls (NC). All miRNAs and siRNAs were transfected using a transfection kit (Ribo, China). The final concentration of miR-199a-3p mimic and mimic NC was 50 nM, while miR-199a-3p inhibitor (100 nM) or inhibitor NC (100 nM) was mixed with ADSC-Exos (2 μg/mL) and added to the SMG-C6 cells post-IR. The final concentration for si-Twist1 and si-NC was 100 nM.

### Vector construction and lentiviral infection

To produce lentivirus, the full-length Twist1 cDNA was subcloned into a CMV-MCS-EF1-T2A lentiviral vector (Hanbio, China) and the lentiviral vectors were then co-transfected with the packaging plasmids psPAX2 and pMD2G into 293T cells. Viral supernatants were collected at 48 and 72 h post-transfection, followed by ultracentrifugation at 82,700 g for 2 h. To establish the stable Twist1 overexpression (OE-Twist1) cell line, SMG-C6 cells were infected with the obtained lentivirus and selected with puromycin. Overexpression negative control (OE-NC) cell line was also established as the negative control. To verify the effect of Twist1 in the IR-injured epithelial cells, SMG-C6 cells post-IR were seeded in 6-well plates and divided into three groups: NC group (OE-NC), Twist1 group (OE-Twist1), Twist1+Exo group (OE-Twist1+Exo).

### Western blot

Cellular proteins were extracted by lysing the cells with RIPA lysis buffer (Millipore, USA), followed by centrifugation at 12,000 g and 4 °C for 15 min to separate the proteins. The protein concentration in each group was determined using a BCA kit (CW0014S, Cwbio, China). Subsequently, the proteins were separated by 12% sodium dodecyl sulfate-polyacrylamide gel electrophoresis (SDS-PAGE) and transferred onto polyvinylidene fluoride (PVDF) membranes (Millipore, USA). The membranes were then blocked and incubated overnight at 4 °C with primary antibodies including rabbit anti-Collagen1 (1:1000, AF7001, Affinity), rabbit anti-TGFβ1 (1:1000, 21898-1-AP, Proteintech, 1:500, GB11179-50, Servicebio), rabbit anti-Twist1 (1:500, 25465-1-AP, Proteintech), rabbit anti-AQP5 (1:1000, 20334-1-AP, Proteintech), rabbit anti-Snail1(1:1000, 13099-1-AP, Proteintech), rabbit anti-CD44 (1:1000, DF6392, Affinity, 1:1000, 60224-1-lg, Proteintch), rabbit anti-Smad3 (1:1000, R25743, Zenbio, China), rabbit anti-p-Smad3 (1:1000, R22919, Zenbio, China) and mouse anti-E-cadherin (1:5000, 60335-1-Ig, Proteintech). Following primary antibody incubation, the membranes were treated with horse-radish peroxidase (HRP)-labeled secondary antibodies: goat anti-rabbit (1:20000, EM35111-01, EMAR) and anti-mouse (1:20000, EM35110-01, EMAR). GAPDH (1:5000, R24404, Zen-Bio) was used as an internal reference, and Image Lab software (Biorad) was used to analyze the relative expression of proteins. The exposure protocol was performed using the Bio-Rad ChemiDoc Imaging System's default auto-exposure model.

### Dual-luciferase reporter assay

The target gene of rno-miR-199a-3p and its binding sequences were predicted and analyzed by TargetScan (https://www.targetscan.org/vert_80/). To further investigate this interaction, luciferase reporter plasmids containing both wild-type and mutated 3' UTR binding sequences of Twist1, a potential target of miR-199a-3p, were constructed. Then, these reporter plasmids were utilized in luciferase activity assays. Briefly, SMG-C6 cells were transfected with recombinant plasmids with either miR-199a-3p mimics or miR-199a-3p-NC, and luciferase activity was assessed using the Dual-Luciferase® Reporter Assay System (11405ES60, Yeasen Biotechnology, China).

### Statistical analysis

SPSS version 19 software was used to perform statistical analysis. All data are presented as mean ± SD. Student's t-test or One-way ANOVA with Turkey's Post-Hoc test was used to analyze differences between groups. Statistical difference was defined as p < 0.05.

## Results

### Identification and characterization of ADSC-Exo

**Figure 1:**
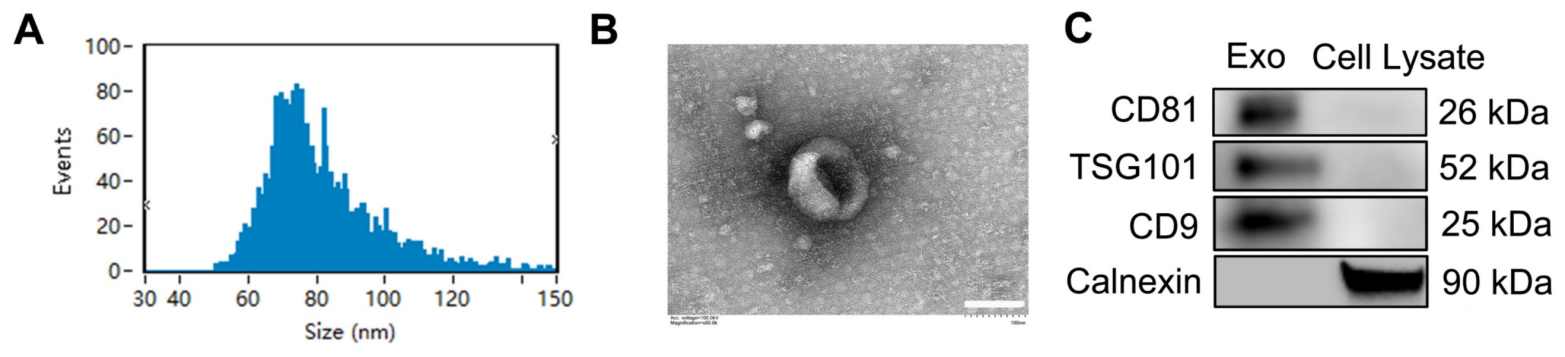
** Identification and characterization of exosomes in conditioned media of ADSCs.** A) Particle size distribution and concentration of ADSC-Exo detected by Nanosight analysis. B) Transmission electron microscopy (TEM) images showing the morphology of ADSC-Exo. Scale bar: 100 nm. C) Western blot analysis of exosome markers (CD81, TSG101, and CD9) and non-exosomal marker (Calnexin) from exosomes and from the control of ADSC lysate.

Nanoparticle tracking analysis (NTA) (Figure 1A) showed that the diameters of the nanoparticles ranged mainly from 50 to 150 nm, with a peak at 70-80 nm. TEM analysis revealed that the exosomes had a cup-shaped morphology and a bilayer membrane structure (Figure 1B). Western blotting results (Figure 1C) confirmed the presence of exosomal markers CD9, CD81 and tumor susceptibility gene 101 (TSG101), and the absence of the non-exosomal marker Calnexin. These findings indicate that ADSC-Exos were successfully isolated.

### ADSC-exosomes restore IR-injured salivary glands *in vivo*

**Figure 2:**
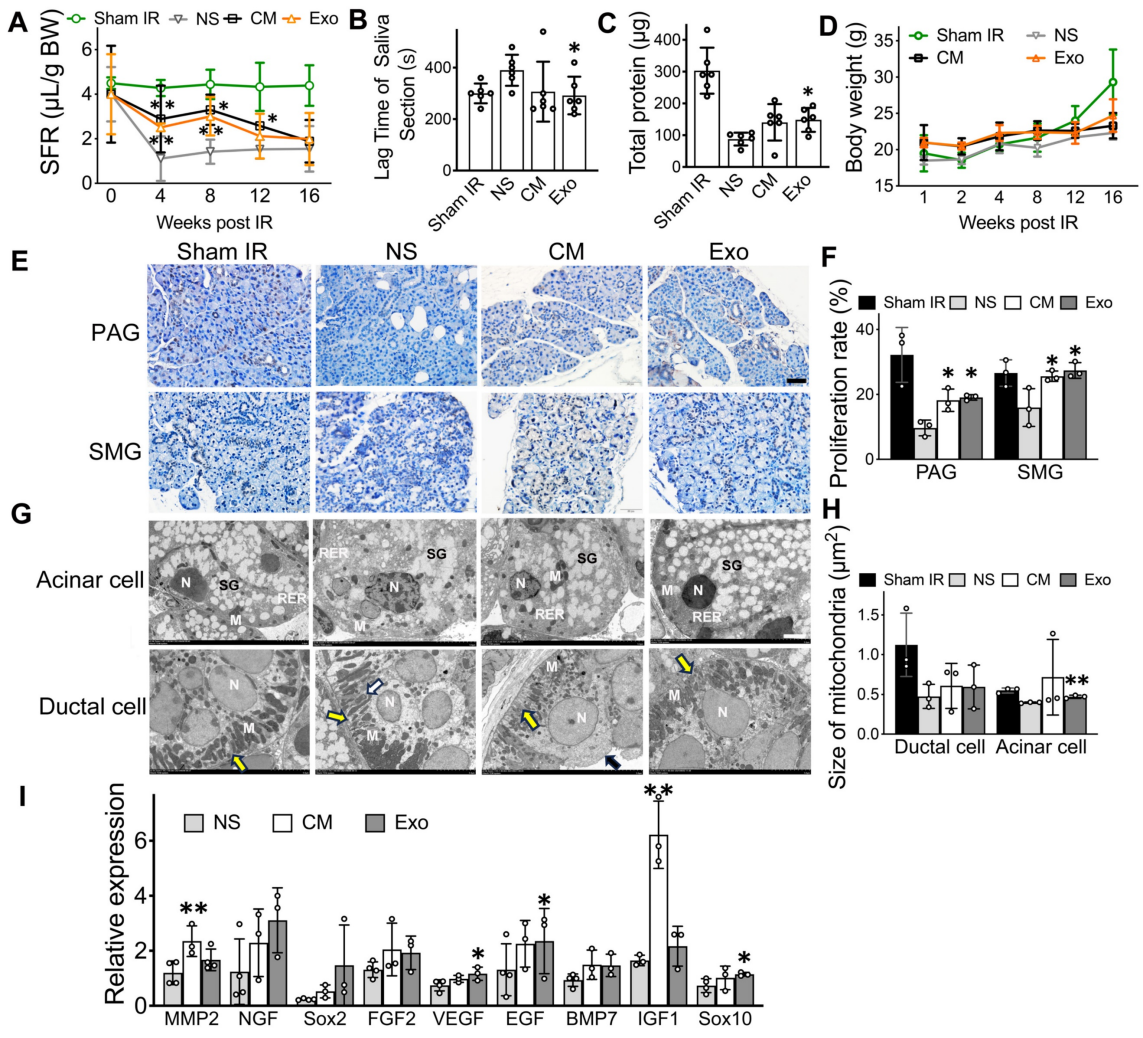
**Exosomes are the active factors in ADSC-CM that restored salivary function to IR-injured SGs *in vivo*.** A) Salivary flow rate (SFR, μL/10 min/g body weight) was measured at week 0 (pre-IR), and weeks 4, 8, 12, and 16 post-IR. B) Time to salivation (lag time) was measured at weeks 8 post-IR. C) Total protein (μg/10 min) in saliva was measured using a BCA assay kit at week 8 post-IR. D) Body weight (g) of the mice. E-F) Ki67 immunohistochemistry staining was used to calculate the salivary cell proliferation rate at week 8 post-IR. Five to eight images were analyzed per sample using ImageJ software (NIH). Scale bar: 50 µm. G) TEM images of acinar and ductal cells. Acinar cells in the Sham IR group show an open-faced nucleus (N), parallel arrays of rough endoplasmic reticulum (RER) located adjacent to the basal membrane, and variable-sized electron lucent secretory granules (SG) occupying the apical cytoplasmic region. Mitochondria (M) with well-developed cristae were also observed in the Sham IR group. In the NS (normal saline) group, acinar cells exhibit an irregular nuclear membrane (N), dilated RER cisternae, secretory granules with disrupted membranes, and numerous coalescent electron secretory granules (SG). These changes are absent in the treated groups. Ductal cells in the Sham IR group show an open-faced, round nucleus (N) and variable-sized mitochondria (M) longitudinally lined in the vertical folding of basal membrane (yellow arrow). In the NS group, ductal cells display a thickened nuclear envelope (N), disorganized basal membrane folds (yellow arrow), and swollen, degenerated mitochondria with cavitation (white arrow). Treated groups show a round nucleus (N), orderly arranged prominent basal membrane folds (yellow arrow), and numerous mitochondria. Microvilli are visible on the luminal surface in the CM group (black arrow). Scale bar: 3 µm. H) Quantification of mitochondria size in ductal cell and acinar cell was analyzed using Image J software (NIH). I) Relative gene expression related to SG repair and regeneration was determined by quantitative real-time PCR. GAPDH served as the endogenous reference. Three experimental replicates were performed for each sample. All data were presented with mean ± SD; * p < 0.05, ** p < 0.01, compared to the NS group (vehicle control group) (n = 3-6).

One of the main findings in this study was that ADSC-Exo was effective in repairing IR-injured SG. As a positive control, human ADSC-CM was used, given its proven success in previous studies for restoring salivary function. By measuring salivary flow rates (SFR), we found that mice in both ADSC-CM and ADSC-Exo groups had their salivary secretory functions partially restored, while IR-mice injected with saline (negative control group) did not (Figure 2A, p < 0.05). Exo-treated mice had a 97.0% and a 59.2% increase, respectively, in SFR at 8- and 12-weeks post-IR when compared to saline-injected mice. To further assess salivary function, the lag time to salivation was measured by observing the first drop of saliva after pilocarpine injection. CM- and Exo-treated mice had a shorter lag time compared to saline-injected IR-mice (Figure 2B, p < 0.05). The total protein in saliva was higher in the Exo-treated group than in the control group (Figure 2C, p < 0.05). Additionally, CM- and Exo-treated mice maintained higher body weights compared to NS-treated mice (Figure 2D, p > 0.05), indicating that CM and Exo injections did not negatively impact body weights.

Histological studies were performed to correlate with salivary function measurements. The cell proliferation rate was significantly higher in the CM and Exo groups than in the NS group (Figure 2F, p < 0.05). Transmission electron microscopy (TEM) was used to observe the ultrastructure of acinar and ductal cells in the salivary gland following the administration of CM and Exo. The average size of mitochondria in the acinar cells was significantly larger in the Exo groups (0.473 ± 0.017 μm^2^) than that of the NS group (0.372 ± 0.014 μm^2^, p < 0.05), while no significant changes were detected in ductal cell (Figure 2H).

We also quantified mRNA levels in mouse salivary tissues using qRT-PCR. When compared to the NS group, the gene expression of MMP2 and IGF1, which are related to SG repair and development, increased by 2-fold and 3-fold, respectively, in CM-treated mice (Figure 2I, p < 0.05). In the Exo-treated group, the gene expression of SG repair and development (EGF) and genes involved in blood vessel and nerve repair/regeneration (VEGF and Sox10) were upregulated (Figure 2I, p < 0.05). Collectively, these data demonstrated that Exo and CM protected functional cells in IR-injured salivary glands, promoted cell proliferation, upregulated tissue regeneration-related gene expression, and restored salivary gland function *in vivo*.

### ADSC-exosomes mitigate epithelial-mesenchymal transition (EMT) in irradiated-injured salivary glands

**Figure 3:**
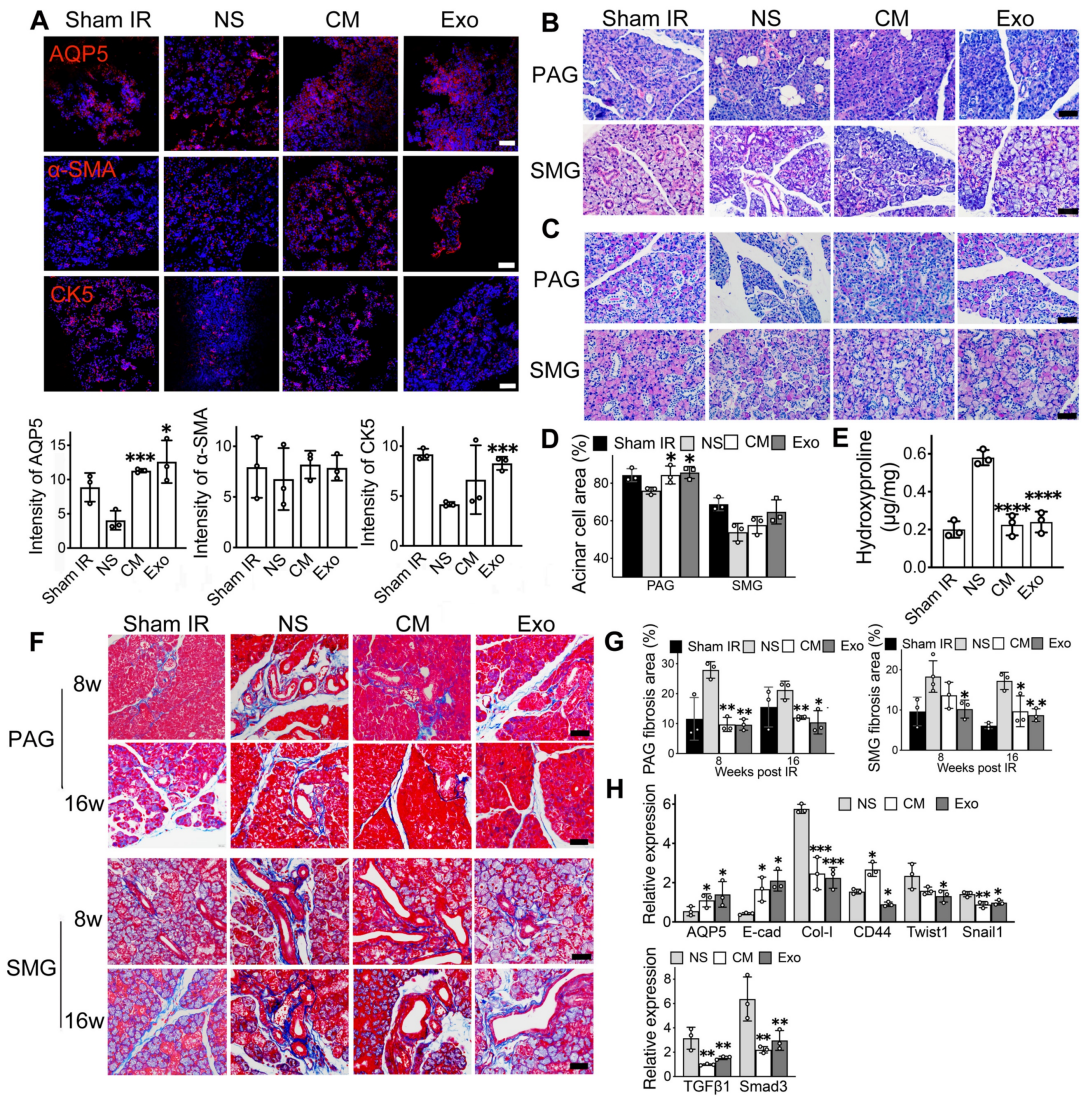
**ADSC-exosomes mitigate epithelial-mesenchymal transition (EMT) in irradiated-injured salivary glands.** A) Immunofluorescent staining of mouse submandibular glands (SMG). Positive cells for AQP5 (acinar cell marker), α-SMA (myoepithelial cell marker), and CK5 (basal ductal cell and some myoepithelial cell marker) were detected on frozen sections. Scale bar: 90 µm. Semi-quantification of immunofluorescent expression for all the markers was analyzed by ImageJ software. Six fields/gland. B) H&E staining of mouse parotid glands (PAG) and submandibular glands (SMG) at 8 weeks post-IR (n = 3). Scale bar: 60 µm. C-D) PAS staining of mouse parotid glands (PAG) and submandibular glands (SMG) (n = 3). Specimens were harvested at 8 weeks post-IR. Scale bar: 60 µm. Quantification of acinar cell area in PAG and SMG was analyzed using ImageJ software (NIH). E) Quantification of total hydroxyproline/collagen (expressed as hydroxyproline (µg) per gland weight (mg)) in SMG (n = 3). F-G) Masson staining of mouse PAG and SMG. Specimens were harvested at 8- and 16-weeks post-IR. Scale bar: 50 µm. Quantification of collagen area in PAG and SMG was analyzed using ImageJ software (NIH). H) Relative expression of fibrosis-related genes determined by quantitative real-time PCR. GAPDH was used as the endogenous reference. All data were presented with mean ± SD; * p < 0.05, ** p < 0.01, *** p < 0.001, **** p < 0.0001 compared to the NS group (vehicle control group) (n = 3). One-way ANOVA with Tukey's Post-Hoc test was used to determine statistical differences among groups.

Immunofluorescent staining was used to identify functional cells in the salivary gland, including acinar cells (AQP5), ductal basal cells (CK5), and myoepithelial cells (α-SMA). Exosome treatment resulted in a 3-fold increase in the relative fluorescent intensity of acinar cells (AQP5) and a 2-fold increase in ductal cells (CK5) compared to NS-treated mice (Figure 3A, p < 0.05). Similarly, CM-treated mice showed higher numbers of AQP5- and CK5-positive cells (Figure 3A). The expression of the α-SMA marker was slightly increased in both CM and Exo groups compared to the NS control group (Figure 3A, p > 0.05). H&E, PAS, and Masson's trichrome staining were conducted to assess the histology of the SMG and PAG, focusing on the surface area of functional cells and collagen-containing connective tissue in the SG. PAS staining showed a significant increase in the acinar cell area in the Exo group within the parotid gland (85.68% ± 3.20%) compared to the NS group (76.00% ± 1.84%, p < 0.05). Although Exo treatment did not significantly increase acinar cell area in the submandibular gland, a trend toward an increase was noted (64.75% ± 6.41% in Exo vs. 53.88% ± 4.73% in NS, p = 0.077, Figure 3C-D).

Masson's trichrome staining revealed significant areas of collagen-containing connective tissue in the SMG of NS-treated mice (Figure 3F). Quantitative analysis showed that the Exo group exhibited significantly less collagen at both 8- and 16-weeks post-treatment (9.61% ± 2.36% in week 8 and 12.03% ± 0.618% in week 16 in PAG, 10.7% ± 1% in week 8 and 8.37% ± 0.97% in week 16 in SMG) when compared to the NS group (27.9% ± 2.77% in week 8 and 21.22 ± 2.96% in PAG, 20.1% ± 3.7% in week 8 and 16.16% ± 1.94% in week 16 in SMG) (Figure 3G, p < 0.05). To quantify total collagen deposition in SMG 8 weeks post-IR, a Hydroxyproline Assay was performed. The results demonstrated a significant increase in collagen deposition in the NS group (0.58 ± 0.04) compared to the CM and Exo groups (0.23 ± 0.05 and 0.24 ± 0.05, respectively) (Figure 3E, p < 0.001), consistent with the findings using Masson's trichrome staining. qRT-PCR analysis demonstrated that Exo treatment influenced the expression levels of genes associated with EMT in the SMG of mice (Figure 3H). The expression of E-cad (an epithelial marker) was 5-fold higher in the Exo treatment group compared to the NS group (p < 0.05). The expression of AQP5 (the acinar cell marker) was a 2.6-fold increase in the Exo group (p < 0.05). Conversely, the expression of fibrotic markers such as Type I collagen (Col-1), CD44, Twist1, and Snail1 was significantly reduced in the Exo treatment group (p < 0.05). The expression of markers in the TGFβ1/Smad3 pathway was 2-fold downregulated in the Exo group (p < 0.05).

**Figure 4:**
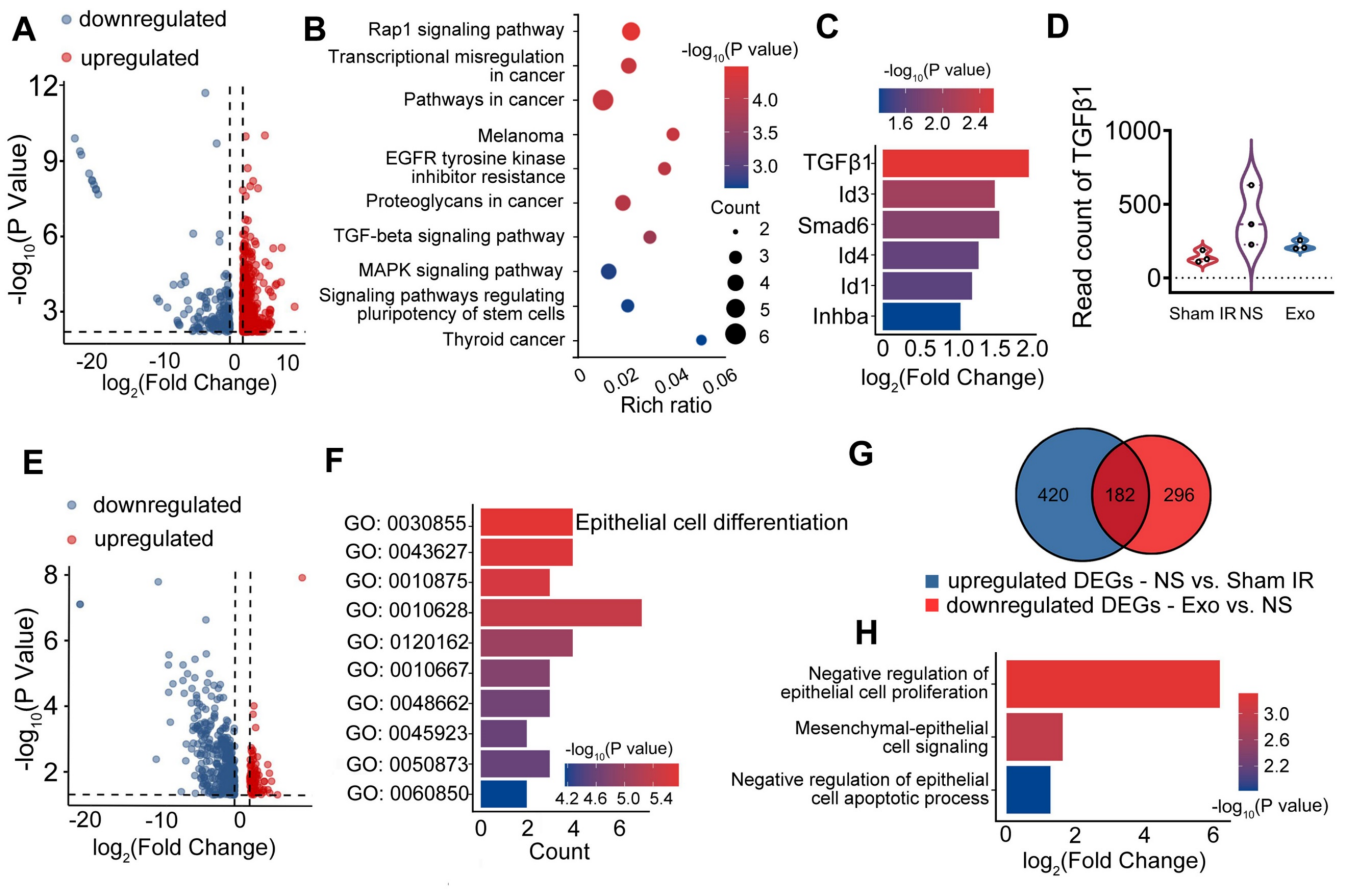
**EMT-related differentially expressed genes (DEGs) and associated signaling pathways.** A) DEGs in the NS (normal saline) group compared to the Sham IR group. B) Kyoto Encyclopedia of Genes and Genomes (KEGG) analysis of upregulated DEGs in the NS group compared to the Sham IR group. C) DEGs related to the TGFβ signaling pathway. D) Read count of TGFβ1. E) DEGs in the Exo group compared to the NS group. F) Gene Ontology (GO) analysis of upregulated DEGs in the Exo group compared to the NS group. G) Venn diagram showing the number of upregulated DEGs in the NS group compared to the Sham IR group and the downregulated DEGs in the Exo group compared to the NS group. H) GO analysis of the overlapping DEGs from the Venn diagram.

RNA sequencing (RNA-seq) was performed to preliminarily profile differentially expressed genes (DEGs) in IR-SG with or without Exo treatment. DEGs were analyzed by the Kyoto Encyclopedia of Genes and Genomes (KEGG) and Gene Ontology (GO) analysis and filtered using the criteria of |log_2_FC| > 1 and p < 0.05. To elucidate DEGs in IR-SG, we profiled the up- and down-regulated DEGs in the NS group compared to the Sham IR group (Figure 4A). The TGFβ signaling pathway was enriched among the upregulated DEGs in the NS group (Figure 4B). DEGs related to the TGFβ signaling pathway were selected (Figure 4C). As a major member of the TGFβ family, TGFβ1 was significantly upregulated in the NS group compared to the Sham IR group (Figure 4C), and its expression in the NS group was also higher when compared to the Exo group (Figure 4D).

To determine the effects of Exo treatment, DEGs of the Exo group were compared to those of the NS group. Upregulated genes in the Exo group are shown as red dots, while downregulated genes are shown as blue dots in the volcano plot (Figure 4E). The upregulated DEGs were enriched in pathways related to epithelial cell differentiation, which was identified as the most significant pathway among the top 10 pathways (Figure 4F). In the Venn diagram, the blue gene set (420 DEGs) represented the upregulated genes in the NS group when compared to the Sham IR group. The red gene set comprised 296 DEGs that were downregulated in the Exo group when compared to the NS group (Figure 4G). The 182 overlapping DEGs from these comparisons were enriched in EMT and epithelial cell proliferation-related biological processes, as revealed by GO enrichment analysis, which included epithelial cell proliferation, mesenchymal-epithelial cell signaling, and epithelial cell apoptotic process (Figure 4H). Taken together, these data suggest that IR promoted the process of EMT, and that Exo treatment regulated EMT-related gene and protein expression and protected salivary functional cells from IR-induced EMT mainly through the TGFβ1/Smad3 pathway.

### ADSC-exosomes inhibit epithelial-mesenchymal transition *in vitro*

**Figure 5:**
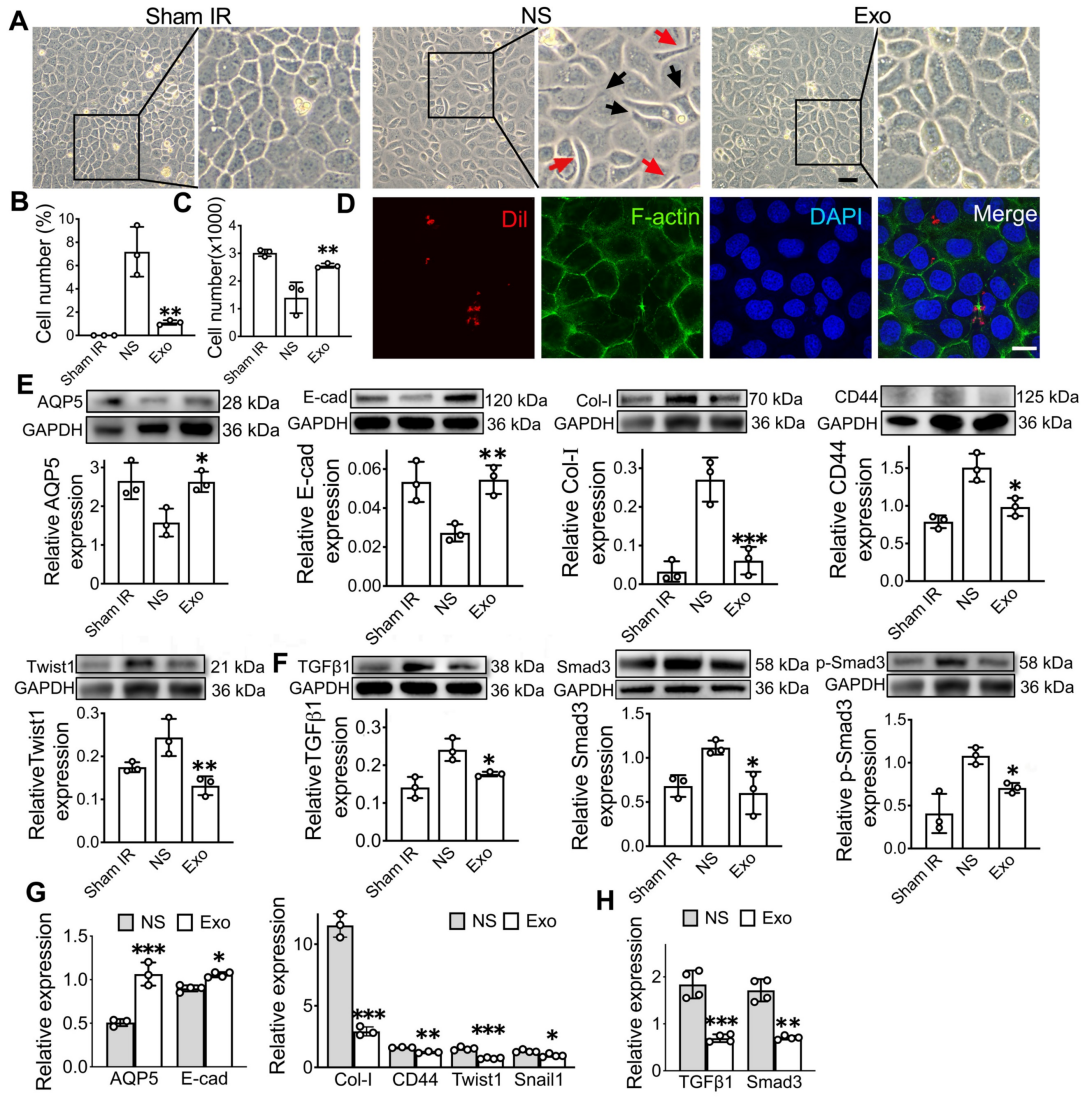
**ADSC-exosome inhibition of epithelial-mesenchymal transition *in vitro*.** A) Morphology changes of the SMG-C6 cells post-IR for 48 h. Elongated, spindle-shaped cells (red arrows) and the pseudopodia (black arrows) were observed in zoomed-in images. Scale bar: 100 μm. B) Quantification of the elongated spindle-like cells in SMG-C6. C) The number of SMG-C6 cell was measured by using a CCK8 kit. NS (normal saline) or ADSC-Exo was added to SMG-C6 group and observed for 48 h. D) Confocal fluorescence analysis showing exosome uptake by SMG-C6 cells. Exosomes were labeled with Dil (red), and the nuclei and cytoskeleton of SMG-C6 cells were stained with DAPI (blue) and F-actin (green). Laser intensity: 0.2% (DAPI), 0.1% (F-actin), 0.4% (Dil). Scale bar: 20 µm. E-H) Expression of epithelial-mesenchymal transition-related proteins/genes (AQP5, E-cad, Col-I, CD44 and Twist1) and TGFβ1/Smad3 pathway-related proteins/genes (TGFβ1, Smad3 and p-Smad3) were tested by WB and qRT-PCR. Three to four experimental replicates were performed for each sample. Data are presented as mean ± SD; * p < 0.05, ** p < 0.01, *** p < 0.001, **** p < 0.0001 compared to the NS group (vehicle control group) (n = 3-4).

Following a single dose of IR, SMG-C6 cells displayed significant morphological changes, transitioning from a typical cobblestone-like shape to an elongated fibroblast-like shape with extended pseudopodia at 48 h post-IR when compared to cells in the Sham IR group (Figure 5A). However, in the Exo group, the number of elongated spindle-like cells was significantly decreased compared to the NS group (Figure 5B, p < 0.01). The effect of Exo on an epithelial cell line was verified *in vitro* by assessing cell proliferation using the CCK8 assay. The results demonstrated a significantly higher cell count in the Exo-treated group (2555 ± 83) compared to the NS group (1397 ± 559, p < 0.05) at 48 h post-irradiation (Figure 5C). To assess whether ADSC-Exo can be internalized by target cells, SMG-C6 cells were incubated with Dil-labeled exosomes. The results confirmed successful internalization, with Dil-labeled exosomes (red) primarily localized within the cytoplasm (Figure 5D). EMT-related protein and gene expressions were analyzed by western blot and qPCR (Figure 5G-H). The gene and protein expressions of epithelial cell markers (AQP5 and E-cadherin) were increased in the Exo group, while the expression of mesenchymal markers (CD44, Collagen1) and EMT-TF related markers (Twist1) were decreased when compared to the NS group (Figure 5E, G, p < 0.05). Additionally, we investigated the effects of exosome treatment on the TGFβ1/Smad3 pathway. The results showed that exosome treatment notably decreased TGFβ1 levels and induced dephosphorylation of Smad3, leading to inactivation of the TGFβ1/Smad3 pathway (Figure 5F, H).

### Exosomal miR-199a-3p alleviates EMT via TGFβ1/Smad3 pathway

**Figure 6:**
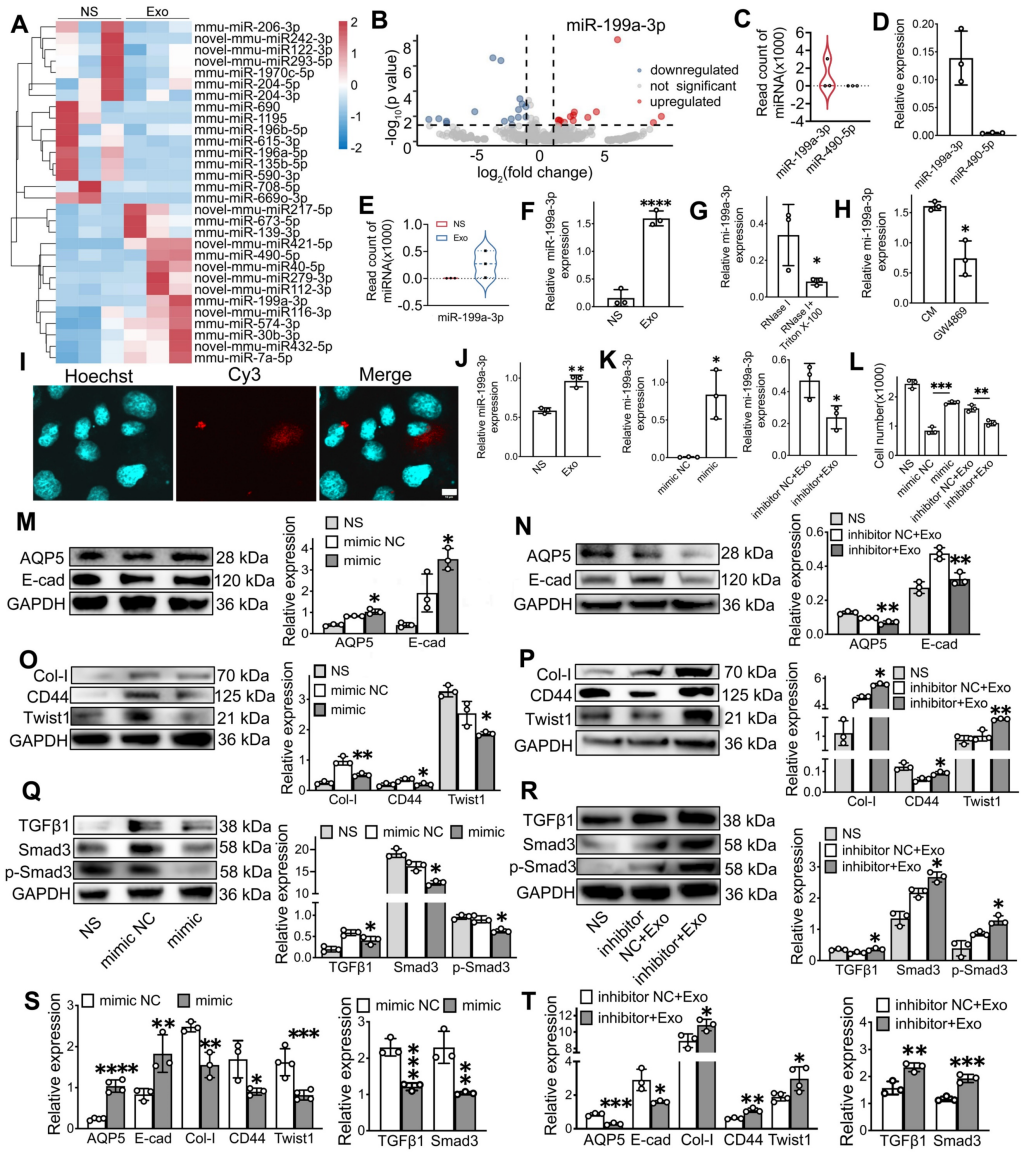
** MiR-199a-3p plays a key role in regulating the IR-SG.** A) Heat map showing the expression levels of microRNAs in SG. B) Volcano plot of microarray showing fold change of differentially expressed microRNAs with criteria of |log_2_FC| > 1, p < 0.05. C-D) Relative gene expression of miR-199a-3p and miR-490-5p in ADSC-Exo by RNA-seq (C) and qPCR (D). miR-484 and U6 were used as endogenous reference. E-F) Relative gene expression of miR-199a-3p in SMG with or without Exo treatment by RNA-seq (E) and qPCR (F). G-H) Relative gene expression of miR-199a-3p in ADSC-CM with different treatments. * p < 0.05, compared to the RNase I or CM group. I) Co-localization of the Cy3-labeled guide strand of miR-199a-3p encapsulated in exosomes with SMG-C6 cells. miR-199a-3p is labeled with Cy3 (red), SMG-C6 cell nuclei are stained with Hoechst (blue). Laser intensities: 31.5% for Hoechst, 63.8% for Cy3. Scale bar: 10 µm. J) Relative gene expression of miR-199a-3p in SMG-C6 cells post-IR with or without Exo treatment. ** p < 0.01, compared to the NS group. K) Relative gene expression of miR-199a-3p in SMG-C6 cells with different treatments. * p < 0.05, compared to the mimic NC or inhibitor NC+Exo group. L) CCK8 was used to analyze the cell number of SMG-C6 post-IR with miR-199a-3p mimic NC (negative control), mimic, inhibitor NC+Exo, inhibitor+Exo or NS (normal saline) treatment. ** p < 0.01, *** p < 0.001. N-T) Relative gene and protein expression of epithelial-mesenchymal transition-related markers (AQP5, E-cad, Col-I, CD44 and Twist1) and TGFβ1/Smad3 pathway-related proteins/genes (TGFβ1, Smad3 and p-Smad3) were tested by WB (M-R) and qRT-PCR (S-T). M, O, Q, S) * p < 0.05, ** p < 0.01, *** p < 0.001, compared to the mimic NC group. N, P, R, T) * p < 0.05, ** p < 0.01, *** p < 0.001, **** p < 0.0001, compared to the inhibitor NC+Exo group. All data were presented with mean ± SD; (n = 3-4).

To investigate the mechanism of exosome treatment, we performed RNA sequencing (RNA-seq) to profile miRNA changes in the SMG, followed by qPCR validation, including miR-199a-3p, miR-217-5p, miR-490-5p, and miR-279-3p, were upregulated, while 16 miRNAs, such as miR-615-3p, miR-135b-5p, and miR-196a-5p, were downregulated in the Exo group compared to the NS group (Figure 6A). Among these, miR-199a-3p showed the most significant difference in expression between the Exo and NS groups (Figure 6B). To identify the effective miRNA in Exo, the expression of the top 5 upregulated miRNAs was tested, but only two miRNAs (miR-199a-3p and miR-490-5p) were detected in ADSC-Exo by both RNA-seq (Figure 6C) and qPCR (Figure 6D). Further qPCR analysis showed that miR-199a-3p levels significantly increased post-Exo treatment in IR-SG *in vivo* (Figure 6E-F, p < 0.05). To determine the origin of miR-199a-3p in ADSC-Exo, we analyzed the levels of miR-199a-3p in CM using qPCR after different treatments. Following the removal of extracellular free miRNA by RNase I, CM pretreated with RNase I and Triton X-100 showed significantly decreased levels of miR-199a-3p compared to CM treated with RNase I alone (Figure 6G). Inhibition of exosome release using GW4869 also led to a marked reduction in miR-199a-3p levels in the treated CM compared to untreated CM, confirming that miR-199a-3p is present in ADSC-derived exosomes (Figure 6H). To visualize the localization of exogenously introduced miR-199a-3p, Cy3-labeled 3'-end double-stranded miR-199a-3p was incorporated into ADSC-Exo. Results showed the exosomal Cy3-labeled miR-199a-3p was successfully delivered to SMG-C6 cells (Figure 6I). Furthermore, qPCR analysis showed that miR-199a-3p levels significantly increased post-Exo treatment in IR-SMG-C6 cells (Figure 6J, p < 0.01) compared to the untreated group. To confirm the effect of exosomal miR-199a-3p on IR-injured SG epithelial cells, miR-199a-3p mimic and inhibitor were introduced to SMG-C6 cells post-IR. The transfection efficiency was confirmed by assessing miR-199a-3p expression levels across treatment groups via qPCR (Figure 6K). The results showed a significant increase in cell number post-IR in the miR-199a-3p mimic group (1800 ± 52) compared to the mimic NC group (847 ± 118, p < 0.05). Conversely, cell numbers decreased in the inhibitor+Exo group (1104 ± 89) compared to the inhibitor NC+Exo group (1607 ± 119, p < 0.05), highlighting the role of exosomal miR-199a-3p in facilitating cell recovery post-IR (Figure 6L).

Further investigation into the effect of miR-199a-3p on EMT in SMG-C6 cells revealed that the ability of exosomes to alleviate IR-induced EMT was modulated by the addition of miR-199a-3p mimics and inhibitors. Specifically, the protein expressions of epithelial cell markers (E-cad, AQP5) significantly increased in the mimic group compared to mimic NC group (Figure 6M, p < 0.05) and decreased in the inhibitor+Exo group compared to the inhibitor NC+Exo group post-IR in SMG-C6 cells (Figure 6N, p < 0.05). Conversely, the expression of mesenchymal-related markers (Collagen1 and CD44) and EMT transcription factor (Twist1) decreased in the mimic group post-IR (Figure 6O, p < 0.05) and increased in SMG-C6 cells treated with Exo and inhibitor post-IR compared to their NC groups (Figure 6P, p < 0.05). The gene expression of epithelial cell and EMT-related markers showed a similar trend to the protein expression results (Figure 6S-T, p < 0.05). To further explore the molecular mechanism of miR-199a-3p in regulating IR-induced EMT, the expression of TGFβ1/Smad3 pathway-related molecules was tested by WB and qPCR. Results demonstrated that miR-199a-3p overexpression in SMG-C6 cells markedly decreased the signals of TGFβ1, Smad3 and p-Smad3 following IR (Figure 6Q, S, p < 0.05), while the signals of TGFβ1, Smad3 and p-Smad3 were increased in the SMG-C6 cell with miR-199a-3p inhibitor adding (Figure 6R, T, p < 0.05). Taken together, our results demonstrated that miR-199a-3p derived from exosomes may play a crucial role in treating IR-induced SG damage by modulating the fibrosis-related pathway (TGFβ1/Smad3) and regulating EMT.

### Twist1, a direct target of miR-199a-3p, regulates IR-EMT via the TGFβ1/Smad3 pathway

**Figure 7:**
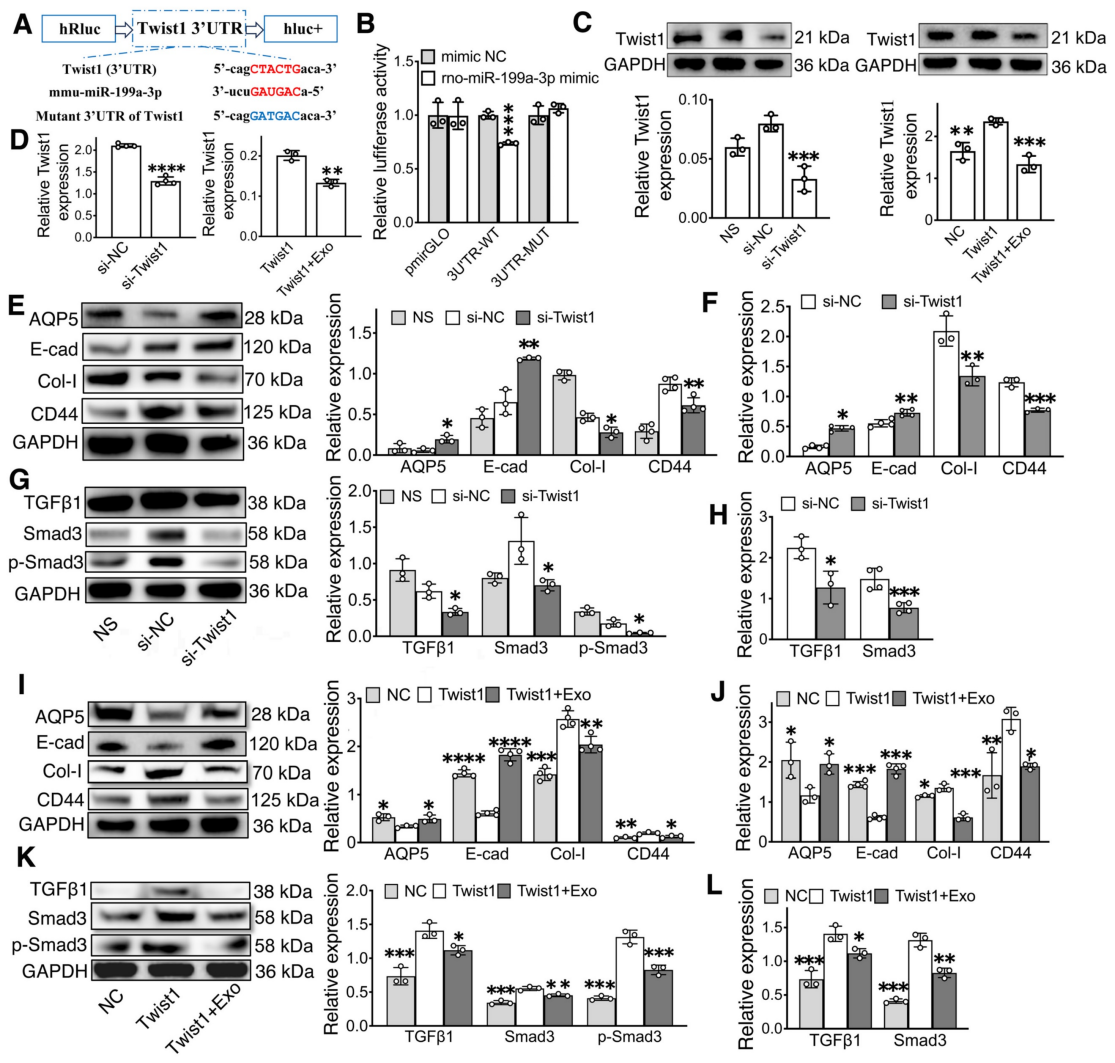
** MiR-199a-3p targets Twist1 and relieves IR-EMT via the TGFβ1/Smad3 pathway.** A) Potential binding sites of miR-199a-3p on the 3' UTR of Twist1. B) Dual-luciferase reporter assay. *** p < 0.001, compared to the mimic NC (negative control) group. C-D) Relative gene and protein expression of Twist1 was determined by qPCR and WB. ** p < 0.01, *** p < 0.001, **** p < 0.0001, compared to the si-NC or Twist1 group. E-L) Relative gene and protein expression of epithelial-mesenchymal transition-related markers (AQP5, E-cad, Col-I, CD44) and TGFβ1/Smad3 pathway-related proteins/genes (TGFβ1, Smad3 and p-Smad3) were tested by WB and qPCR. * p < 0.05, ** p < 0.01, *** p < 0.001, **** p < 0.0001, compared to the si-NC or Twist1 group. All data were presented with mean ± SD; (n = 3-4).

To further explore the molecular mechanism of miR-199a-3p in regular EMT, the possible targets of miR-199a-3p were predicted on the publicly available database (Targetscan). Based on bioinformatics analysis, Twist1 was selected as a target gene of miR-199-3p (Figure 7A). The direct interaction between miR-199a-3p and Twist1 was confirmed by a dual-luciferase reporter assay. The miR-199a-3p mimic significantly suppressed the luciferase activity of the reporter gene with the wild-type 3'UTR of Twist1. This inhibitory effect was abolished when using the vector containing the Twist1-mutated 3'UTR (Figure 7B, p < 0.05). Additionally, compared to the NC group, Twist1 expression significantly decreased in the miR-199a-3p mimic group and increased in the miR-199a-3p inhibitor group (Figure 6K-L, O-P, p < 0.05).

To further investigate the role of Twist1 in regulating EMT and the TGFβ1/Smad3 pathway, we performed knockdown and overexpression experiments of Twist1 in SMG-C6 cells using small interfering RNA (siRNA) and lentivirus. Both Twist1 gene and protein were downregulated in the si-Twist1 group and upregulated in the Twist1 group when compared to their NC groups (Figure 7C-D, p < 0.05). Furthermore, the expression of Twist1 was downregulated in the Twist1+Exo group compared to the Twist1 group (Figure 7C-D, p < 0.05). In addition, western blot and qPCR results showed that the expressions of E-cadherin and AQP5 were significantly increased in the si-Twist1 group and decreased in the Twist1(Twist1 overexpression) group compared to the NC (control) groups (Figure 7E-F, I-J, p < 0.05). Conversely, the expressions of Collagen I and CD44 showed the opposite trend (Figure 7E-F, I-J, p < 0.05). Moreover, underexpressing Twist1 markedly attenuated IR-induced activation of TGFβ1, Smad3 and phosphorylation of Smad3, while overexpressing Twist1 enhanced the activation of the TGFβ1/Smad3 pathway compared to their control groups (Figure 7 G-H, K-L, p < 0.05). A Twist1+Exo group was also included to further verify the effect of exosome in mitigating EMT by regulating Twist1 expression. Our results showed that the expressions of epithelial markers (E-cad and AQP5) were significantly increased, while the expressions of mesenchymal markers (Col-I and CD44) and TGFβ1/Smad3 pathway-related markers were decreased in the Twist1+Exo group when compared to the Twist1 overexpression group (Figure 7I-L, p < 0.05).

## Discussion

Adipose-derived stem cells are a minimally invasive cell source that can be isolated from a plethora of adipose tissues, making them ideal for clinical applications. Studies showed that ADSCs transplantation reduced the expression of proteins related to fibroblast activation (such as TGFβ, Snail, Twist, and c-Myc), limited EMT and fibrosis, and repaired IR-induced damage to the lungs and salivary glands 13, 39. While stem cell transplantation has shown promise in restoring salivary gland function 40-43, growing evidence suggests that stem cells' paracrine effects are the main mechanism behind their efficacy. This has led to the exploration of cell-free therapies as potentially superior to cell-based therapies. Compared to cell-based approaches, cell-free therapies are easier to extract, more stable for storage, and exhibit lower levels of immunogenicity and toxicity 44.

Conditioned medium, often referred to as the cell secretome, is a widely used cell-free therapy. ADSC-CM helps repair IR-damaged salivary glands by promoting cell growth and salivary protein production 45. However, CM is complex, containing soluble proteins (SP, such as cytokines, chemokines, enzymes, signaling and signal transduction proteins, and cell adhesion molecules), nucleic acids (DNA, RNA, microRNAs), lipids, and extracellular vehicle (EV, encompassing apoptotic bodies, micro-vesicles, and exosomes) 46. To simplify, exosomes stand out as promising bioactive factors due to their simpler composition, stability in circulation, and targeted delivery capabilities. Although the exact fate of Exo in circulation remains unclear, studies indicate that they frequently accumulate in organs such as the spleen, lungs, and kidneys 47. However, other studies demonstrate that exosomes can reach various target organs via intravenous (IV) injection 48-51, including the salivary gland 52, 53. Exosomes also exhibit homing abilities in organs such as the bowel 48, liver 49, heart 50, 54, and brain 51, where they target tumors 55, inflamed tissues 48, and injured organs 49, demonstrating therapeutic benefits via systemic administration 48, 49, 51. Exosomes have been used to treat Sjögren's syndrome via IV injection 30. IV injection is a well-established delivery route with benefits in terms of safety, ease of administration, and reduced invasiveness compared to local injections 50. To enhance the accumulation of exosomes in the SG, we used a higher dose (100 µL), administered twice, which is 3 to 5 times greater than the dose typically used for local injections 35, 56. Our findings demonstrate that ADSC-Exo, delivered via IV injection, effectively treated IR-SG by enhancing saliva secretion, increasing total salivary protein levels, and reducing salivary lag time (Figure 2A-C). Furthermore, Exo treatment significantly promoted cell proliferation in IR-SG (Figure 2E-F). Studies on the ultrastructure of IR-SG are limited. Our research observed morphological changes in the nucleus, rough endoplasmic reticulum (RER), secretory granules, and mitochondria in acinar and ductal cells. The results showed that Exo treatment protected the ultrastructure of both acinar and ductal cells post-IR (Figure 2G). Interestingly, the mitochondria in acinar cells significantly increased in size in the Exo group compared to the NS group, while no significant change was found in ductal cells (Figure 2H). This could be because acinar cells are more sensitive to IR damage and that their repair are more noticeable. In addition, there is an upregulation of gene expression for repair/regeneration in IR-SG treated with Exo (Figure 2I). These results suggest that Exo is a promising therapy for treating IR-SG by protecting functional cells, promoting cell proliferation, and regulating its repair and regeneration.

While Exo and CM treatments demonstrated similar protective trends, their levels of repair differed. For instance, CM treatment protected mitochondria but did not significantly increase mitochondrial size (p > 0.05), potentially due to individual variability. CM treatment also upregulated genes involved in tissue remodeling (MMP2) and neural recovery (IGF1), whereas Exo therapy significantly upregulated genes associated with angiogenesis (VEGF) and epithelial cell repair (EGF and Sox10) (Figure 2I). Interestingly, CD44 expression varied between treatments. CD44 was upregulated in the CM group while downregulated in the Exo group, when compared to the NS vehicle control (Figure 3H). CD44, a multifunctional glycoprotein and receptor for MMPs, is linked to stem cell maintenance, DNA repair, and anti-apoptosis 57, 58, which may contribute to protective effects of CM treatment. However, high CD44 expression may enhance fibroblast activity and EMT in irradiated tissues 58, which are linked to fibrosis progression 59. Thus, reduced CD44 in the Exo group may indicate less EMT activation. Taken together, CM and Exo protect IR-SG through different mechanisms, and further studies are needed to better understand these differences.

As an additional objective, we examined the therapeutic effects of secreted soluble factors (SP) from CM alongside the Exo therapy to identify the main paracrine components in ADSC secretome. Exo and SP are the primary fractions in CM suitable for clinical separation, whereas other components are difficult to isolate and yield lower quantities, limiting their clinical utility. Previous studies have shown distinct roles for SP and Exo. For instance, one study emphases the immunomodulatory role of MSC-derived SP on B cells rather than the extracellular vesicles 60. Another study reported that removing exosomes from CM negated its protective effects in myocardial ischemia injury 61. In this study, SP treatment failed to restore the secretory function and to increase the acinar cell area in IR-SG but did promote cell proliferation and upregulated genes involved in SG repair and regeneration (Figure S1). These findings suggest that SP comprehensively promotes SG repair and regeneration, protecting various cell types in SG, albeit excluding acinar cells. Consequently, our results indicate that the therapeutic benefits of the ADSC secretome are partially mediated by Exo rather than SP, suggesting exosomes as a promising treatment for IR-injured SG.

The process of IR-SG is categorized into acute and chronic stages in mouse and rat models 2. In the chronic stage (≥30 days post-IR), interactions between IR-damaged tissue-resident cells and the surrounding microenvironment trigger signaling pathways that promote excessive extracellular matrix (ECM) deposited, leading to fibrosis 2, 62. Fibrosis typically appears at 4-6 months post-IR in mouse models 2, 5, and as early as 1 month after fractionated IR in mini-pigs 63. Recent studies have found significant fibrosis in IR-injured SG as early as 3 months post-IR in mouse models 42, 64. In line with these findings, fibrosis was detected at 16 weeks post-IR in our study. In addition, our study observed a decline in SG secretory function starting from week 1 post-IR, which continued to decrease and stabilized at lower levels by 4 weeks post-IR. At week 8 post-IR, mesenchymal cell markers were overexpressed, while epithelial cell markers were downregulated in the untreated group (NS group) when compared to the Sham IR group (Figure 3H). Furthermore, both the hydroxyproline assay and Masson's trichrome staining confirmed the occurrence of EMT at 8 weeks post-IR, while Exo treatment significantly reduced collagen deposition in the IR-SG. These findings indicate that EMT was actively occurring within the SG parenchyma during this period and that Exo treatment can mitigate fibrosis in IR-SG.

Current insight into EMT suggests that relying on a few EMT molecular markers is insufficient to evaluate EMT status 65. A comprehensive assessment of EMT-related mRNA and protein levels before and after treatment is crucial 65. In our study, a panel of mesenchymal-related genes and proteins (e.g., Col-1, CD44, Twist1, Snail1) were downregulated in Exo-treated mice, while epithelial markers (E-cadherin, AQP5) were upregulated (Figure 3), indicating that Exo treatment inhibited the EMT process in IR-injured epithelial cells. Interestingly, α-SMA expression was similar between treated and untreated groups (Figure 3A). While α-SMA is a recognized fibrosis marker in IR-injured lung models 13, its role in the SG is controversial. Treatments using ADSC or amifostine lead to SG tissue remodeling with increased expression of α-SMA and a decrease in fibrosis 39, 66. However, elevated α-SMA has been linked to chronic sialadenitis 10 and TGFβ-related fibrosis in the SG 67. This ambiguity arises mainly because α-SMA serves as a biomarker for both myofibroblasts (associated with fibrosis) and myoepithelial cells (essential for SG function) 68. Therefore, α-SMA was not utilized as an EMT marker in our mechanistic study *in vitro*. EMT-associated transcription factors (EMT-TFs), co-expressed in cells, play crucial roles in orchestrating EMT processes 69. We observed a reduction in core EMT-TFs (Twist1, Snail1) with Exo treatment, supporting its inhibitory effect on EMT. To thoroughly evaluate EMT, we investigated SG function and acinar cell percentages *in vivo*, as well as the morphology of salivary epithelial cells *in vitro*. Mice receiving Exo treatment showed improved SG function, with a significant increase in acinar cell area in PAG. Although the increase in SMG was not statistically significant, a positive trend was observed, potentially due to the small sample size and variability in acinar cell percentages between PAG and SMG. Additionally, Exo-treated cells retained an epithelial shape *in vitro*. Moreover, Exo treatment inhibited the TGFβ1/Smad3 signaling pathway, which regulates EMT and fibrosis 8, leading to improved secretory function and reduced fibrosis. In summary, Exo treatment attenuated EMT, partially through the TGFβ1/Smad3 pathway, restoring salivary gland function.

To identify crucial miRNAs involved in Exo treatment, RNA-seq was performed. Our results revealed multiple miRNAs were regulated by exosome treatment in mouse SG, with miR-199a-3p showing the most significant upregulation (Figure 6A-B). Additionally, our results confirm the presence of miR-199a-3p in ADSC-Exo (Figure 6C-H) and demonstrate that miR-199a-3p was effectively delivered to SMG-C6 cells through co-culture with exosomes (Figure 6I). Gene expression analysis showed that miR-199a-3p, which was reduced after IR, significantly increased in IR-SG and IR-SMG-C6 cells following Exo treatment compared to untreated cells (Figure 6E-F, J-L). These findings indicated that miR-199a-3p in Exo might be a primary molecule in treating IR-SG. Exosomal miR-199a-3p has been reported to promote cell cycle re-entry, cell proliferation, suppress ferroptosis 70 and oncogenesis 71, 72, and reduce cell senescence 73, 74 and myocardial fibrosis 25. MiR-199a carried by extracellular vesicle also targets GREM1 to inactivate the TGFβ pathway 75. However, the function of miR-199a-3p in IR-SG injury remains unclear. To clarify this, we transfected an inhibitor and mimic of miR-199a-3p into SMG-C6 cells post-IR. The mimic group showed a doubling of the cell number compared to the control group, while the inhibitor+Exo group showed reduced cell proliferation, confirming the protective effect of exosomal miR-199a-3p on IR-injured epithelial cells (Figure 6L). Further analysis revealed that the miR-199a-3p mimic downregulated pro-fibrotic markers and TGFβ1/Smad3 pathway molecules, while upregulating epithelial markers (Figure 6M-T). These results indicate that miR-199a-3p in Exo attenuated IR-EMT via the TGFβ1/Smad3 pathway.

Nevertheless, the specific molecular target of miR-199a-3p in regulating the TGFβ1/Smad3 pathway and EMT remains unexplored. Therefore, we analyzed the predicted miR-199a-3p targets in KEGG pathway related to fibrosis and identified Twist1 as a novel target through a luciferase reporter assay. Twist1 is a crucial transcription factor that regulates IR-induced fibrosis by facilitating IR-EMT 15. Previous studies have linked Twist1 with the development of IR-induced fibrosis and demonstrated that inhibiting Twist1 can alleviate fibrosis in liver and lung 12, 76. In line with these findings, our study showed that Twist1 inhibition decreased EMT by downregulating mesenchymal markers and upregulating epithelial cell markers in SMG-C6 cells (Figure 7). Additionally, Twist1 has been reported to induce EMT and renal fibrosis via the TGFβ pathway 77. In our study, Twist1 overexpression activated the TGFβ1/Smad3 pathway, while Twist1 knockdown suppressed the activation of TGFβ1, Smad3 and the phosphorylation of Smad3, thereby attenuating the IR-EMT. To verify the effect of Exo on Twist1, Exo treatment was applied on the Twist1-overexpressing cell post-IR (Figure 7C-D, I-L). Results showed that Exo treatment significantly suppressed the EMT by downregulating Twist1 and inactivating the TGFβ1/Smad3 signaling pathway. Together, these findings suggest that miR-199a-3p in Exo plays a role in mitigating IR-induced EMT by targeting Twist1 and regulating the TGFβ1/Smad3 pathway.

In the present study, we observed that the Exo treatment effectively alleviates salivary hypofunction by mitigating fibrosis through the Twist1/TGFβ1/Smad3 pathway. These findings suggest that increased fibrogenic activity contributes to SG hypofunction and addressing EMT could be a promising strategy to restore SG function. This study provides new insights into the understanding of IR-SG injury and proposes a potentially effective treatment for SG hyposalivation. Nevertheless, our study has certain limitations. First, although we detected fibrotic damage at 4 months post-IR in SG, future investigations could benefit from extending the observation period to track the progression of gland fibrosis and assess long-term therapeutic effects. Second, conducting additional animal experiments could further validate the mechanisms underlying exosome treatment. Additionally, exosome injections were administered 5 to 7 days post-IR to minimize oncogenic risk and follow humane practices for irradiated mice. However, given the limited studies on exosome treatment in IR-SG, the optimal timing for this therapy requires further study. Further investigation into the influence of exosome treatment on tumor responses is needed before progressing to clinical applications. Lastly, while miR-199a-3p has shown potential in treating IR-SG by inhibiting EMT, it remains unclear whether other essential factors derived from Exo contribute to this process and whether miR-199a-3p possesses additional therapeutic functions in addressing IR-SG.

## Conclusions

Our study showcased the efficacy of ADSC-Exo in mitigating IR-induced injury to salivary glands, both *in vivo* and *in vitro*. Specifically, miR-199a-3p in Exo targeted Twist1, leading to the downregulation of the TGFβ1/Smad3 signaling pathway and consequently inhibiting IR-induced EMT. This insightful understanding of ADSC-Exo's role in IR-SG enhances the potential for developing new therapeutic interventions with clinical applications.

## Supplementary Material

Supplementary figures and table.

## Abbreviations

α-SMA: alpha smooth muscle actin
ADSC-CM: adipose-derived stem cell-derived conditioned medium
ADSC-Exo: adipose-derived stem cell-derived exosome
ADSCs: adipose derived stem cells
AQP5: aquaporin 5
BCA: bicinchoninic acid
BMP7: bone morphogenetic protein 7
CCK-8: cell counting kit-8
CK5: cytokeratin 5
CM: conditioned medium
DAB: diaminobenzidine
DAPI: 4, 6-diamidino-2-phenylindole, dihydrochloride
DEGs: differentially expressed genes
DNBs: DNA nanoballs
ECM: extracellular matrix
EGF: epidermal growth factor
EMT: epithelial-mesenchymal transition
EV: extracellular vesicle
Exo: exosomes
F-actin: filamentous actin
FGF2: fibroblast growth factor 2
GAPDH: glyceraldehyde-3-phosphate dehydrogenase
GO: gene ontology
H&E: Hematoxylin and Eosin
HRP: horse-radish peroxidase
IF: immunofluorescent
IGF1: insulin-like growth factor 1
IHC: immunohistochemistry
IR-EMT: IR-induced EMT
IR-SG: irradiation-injured salivary glands
IV: intravenous
KEGG: kyoto encyclopedia of genes and genomes
miRNA: microRNAs
MMP2: matrix metalloproteinase 2
MSC: mesenchymal stem cell
NC: negative control
NGF: nerve growth factor
NS: normal saline
NTA: nanoparticle tracking analysis
OCT: optimal cutting temperature
OD: optical density
OE: overexpression
PAG: parotid gland
PAS: periodic acid-schiff
PBS: phosphate buffered saline
PVDF: polyvinylidene fluoride
qRT-PCR: quantitative polymerase chain reaction
RIN: RNA integrity number
RIPA: radio immunoprecipitation assay
RNA-seq: RNA sequencing
RT: radiotherapy
SDS-PAGE: sulfate-polyacrylamide gel electrophoresis
SFR: salivary flow rate
SG: salivary gland
SMG: submandibular gland
SMG-C6: submandibular gland C6
Snail1: snail family transcriptional repressor 1
SP: soluble protein
TEM: transmission electron microscopy
TF: transcription factor
TGFβ1: transforming growth factor β1
TSG101: tumor susceptibility gene 101
VEGF: vascular endothelial growth factor
WB: western blot
