# Supplementary material for: Exosomes of human adipose stem cells mitigate irradiation injury to salivary glands by inhibiting epithelial-mesenchymal transition through miR-199a-3p targeting Twist1 and regulating TGFβ1/Smad3 pathway
Source: Theranostics. 2025 Jan 2;15(5):1622–41. doi: 10.7150/thno.102346 (PMC11780531; doi:10.7150/thno.102346)
Supplement: Supplementary file 1 — Supplementary figures and table. [file thnov15p1622s1.pdf]

Exosomes of human adipose stem cells mitigate irradiation injury to salivary glands by inhibiting epithelial-mesenchymal transition through miR-199a-3p targeting Twist1 and regulating the TGFβ1/Smad3 pathway

Xiaotong Guo[1], Zhu Huang[1], Fan Wu[1], Wentao Jiang[1,3], Yiyang Li[1], Tao Wang[1], Simon D Tran[2], Zhengmei Lin[*1], Xinyun Su[*1]

Supplementary Figures and Table

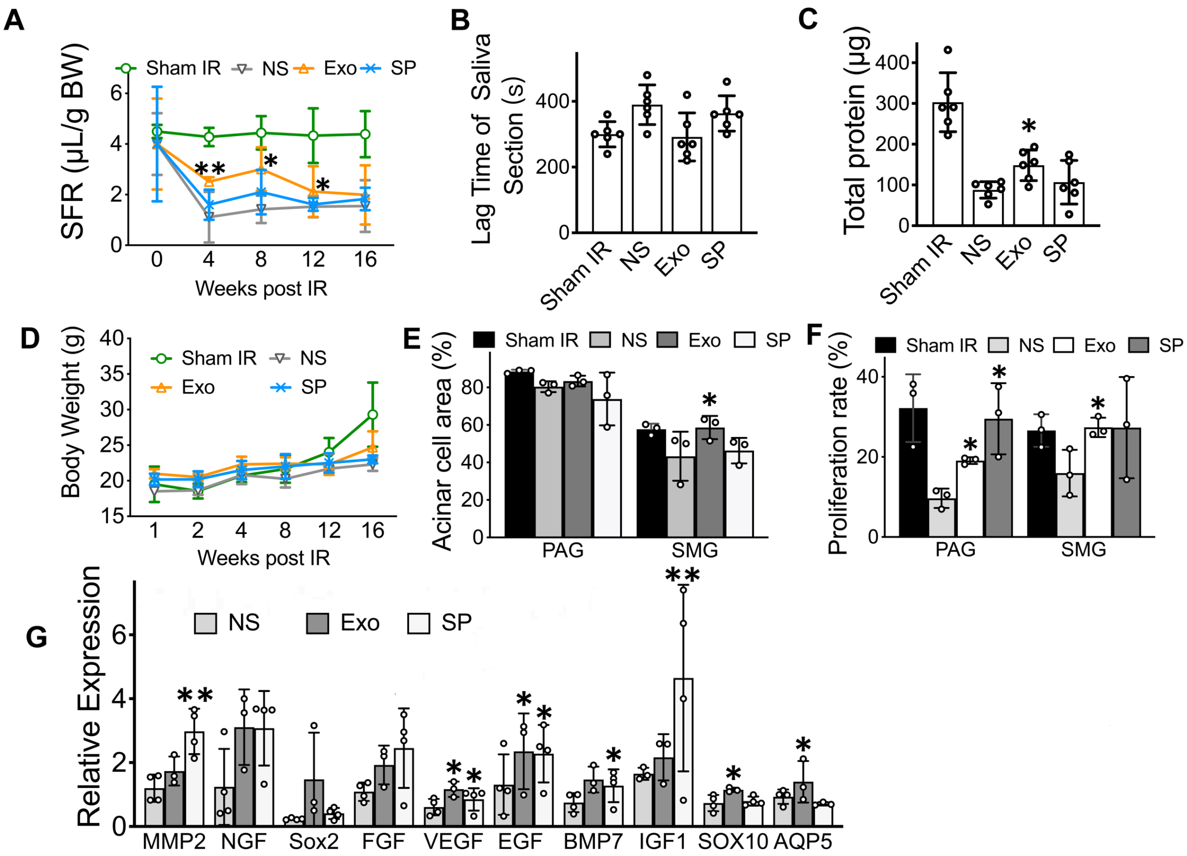

Figure S1. The effect of SP in treating the hypofunction of IR-SG

A) Salivary flow rate (SFR, μL/10 min/g body weight) was measured at week 0 (before IR), 4, 8, 12 and 16 post-IR. B) Time to salivation (lag time) was measured at week 8 post-IR. C) Total protein(μg/10 min) in saliva was measured by a BCA assay kit at week 8 post-IR. D) Body weight (g) of the mouse. E) Quantification of acinar cells area in PAG and SMG according to the H&E staining. F) Ki67 immunohistochemistry staining was used to calculate the salivary cell proliferation rate at week 8 post-IR. G) Relative expression of genes related to SG repair and regeneration was determined by quantitative real-time PCR. GAPDH was used as the endogenous reference. Three experimental replicates were performed for each sample. All data were presented with mean ± SD; * $p < 0.05$, ** $p < 0.01$, compared to the NS group (n = 3-6).

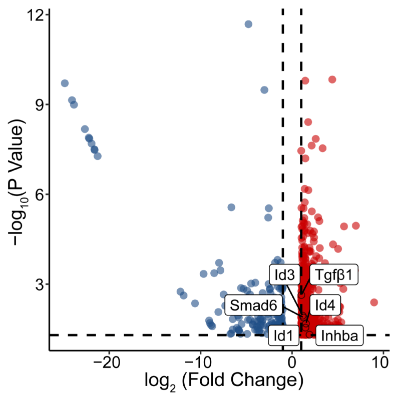

Figure S2.

Kyoto Encyclopedia of Genes and Genomes (KEGG) analysis of TGFβ signaling pathway related DEGs in the NS group compared to the Sham IR group.

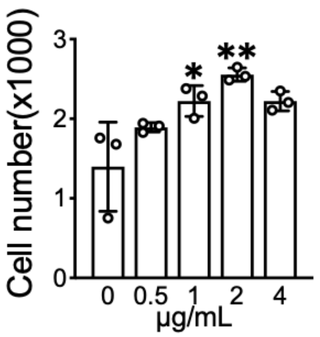

Figure S3. Concentration of Exo treatment in vitro

The number of SMG-C6 cell was measured by using a CCK8 kit. NS or different concentration of ADSC-Exo (0.5, 1, 2 and 4 µg/mL) was added to SMG-C6 cells and observed for 48 h. 2 µg/mL Exo was chosen for the experiments in vitro. All data were presented with mean ± SD; * $p < 0.05$, ** $p < 0.01$, compared to the 0 µg/mL group (n = 3).

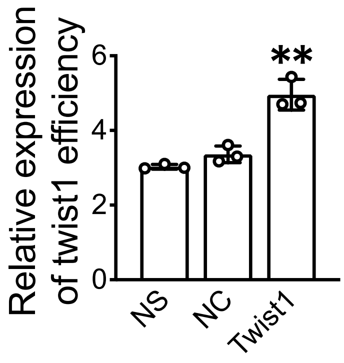

Figure S4. The efficiency of Twist1 infection

Lentivirus infection was used for Twist1 overexpression experiment in SMG-C6 cells. A significant increase of Twist1 expression was detected by qPCR. All data were presented with mean ± SD; ** $p < 0.01$, compared to the NS group ($n = 3$).

Table S1. Primer sequences used for qPCR

genes	primer forward（5’ -3’）	primer reverse（5’ -3’）
mmu-EGF	AGCATCTCTCGGATTGACCCA	CCTGTCCCGTTAAGGAAAACTCT
mmu-VEGF	GCACATAGGAGAGATGAGCTTCC	CTCCGCTCTGAACAAGGCT
mmu-IGF1	CTGGACCAGAGACCCTTTGC	GGACGGGGACTTCTGAGTCTT
mmu-FGF2	GCGACCCACACGTCAAACTA	TCCCTTGATAGACACAACTCCTC
mmu-BMP7	ACGGACAGGGCTTCTCCTAC	ATGGTGGTATCGAGGGTGGAA
mmu-NGF	CCAGTGAAATTAGGCTCCCTG	CCTTGGCAAAACCTTTATTGGG
mmu-MMP2	CAAGTTCCCCGGCGATGTC	TTCTGGTCAAGGTCACCTGTC
mmu-Sox10	ATCAGCCACGAGGTAATGTCCAAC	ACTGCCCAGCCCGTAGCC
mmu-AQP5	TCTACTTCTACTTGCTTTTCCCCTCCTC	CGATGGTCTTCTTCCGCTCCTCTC
mmu-GAPDH	ACTCCACTCACGGCAAATTCAACGGCACAG	GGGTCTCGCTCCTGGAAGATGGTGATGGG
mmu-E-Cadherin	CAGCCGGTCTTTGAGGGATT	TGACGATGGTGTAGGCGATG
mmu-Collagen Ⅰ	AGCACGTCTGGTTTGGAGAG	GACATTAGGCGCAGGAAGGT
mmu-CD44	CATGGAATACACCTGCGTAGC	CTAGATCCCTCCGTTTCATCC
mmu-TGFβ1	TCGACATGGAGCTGGTGAAG	GCAGCTTGGACAGGATCTGG
mmu-Smad3	AGAAGCTCAAGAAGACGGGG	TGACCTGGGGATGGTAATGC
mmu-Twist1	AGCGGGTCATGGCTAACG	ATCTTGCTCAGCTTGTCCGAG
mmu-GAPDH	CATCACTGCCACCCAGAAGACTG	ATGCCAGTGAGCTTCCCGTTCAG
mmu-AQP5	CTCCCCAGCCTTATCCATTG	CCAGAAGACCCAGTGAGAGG
rno-E-Cadherin	ATCCTGGCCCTCCTGATTCT	CGGGTATCGTCATCTGGTGG
rno-Collagen Ⅰ	GAGCGGAGAGTACTGGATCG	TACTCGAACGGGAATCCATC
rno-CD44	CATGGAATACACCTGCGTAGC	CTAGATCCCTCCGTTTCATCC
rno-TGFβ-1	GCAACAACGCAATCTATGAC	GCAGCTTGGACAGGATCTGG
rno-Smad3	AGGAGAAGTGGTGCGAGAAG	GTGACCTGGGGATGGTAATG
rno-Twist1	CTCGGACAAGCTGAGCAAGAT	CTCGTGGGCCACATAGCTG
rno-GAPDH	ATGCTGGTGCTGAGTATGTC	AGTTGTCATATTTCTCGTGG
rno-AQP5	CTGCATCTTCTCCTCCACCG	CCCTACCCAGAAGACCCAGT
miRNA	Primer (5’-3’)	
miR-199a-3p	CGACAGTAGTCTGCACATTGGTTA	
miR-490-5p	CCATTGATCTCCAGGTGGGT	
miR-484	GCTCAGTCCCCTCCCGAT	
U6	CTCGCTTCGGCAGCACA	
